# Predicting Therapy Success and Costs for Personalized Treatment Recommendations Using Baseline Characteristics: Data-Driven Analysis

**DOI:** 10.2196/10275

**Published:** 2018-08-21

**Authors:** Vincent Bremer, Dennis Becker, Spyros Kolovos, Burkhardt Funk, Ward van Breda, Mark Hoogendoorn, Heleen Riper

**Affiliations:** ^1^ Institute of Information Systems Leuphana University Lüneburg Germany; ^2^ Department of Clinical, Neuro- & Developmental Psychology Vrije University Amsterdam Netherlands; ^3^ Department of Health Sciences Vrije University Amsterdam Netherlands; ^4^ Department of Computer Science Vrije University Amsterdam Netherlands

**Keywords:** treatment recommendation, cost effectiveness, mental health, machine learning

## Abstract

**Background:**

Different treatment alternatives exist for psychological disorders. Both clinical and cost effectiveness of treatment are crucial aspects for policy makers, therapists, and patients and thus play major roles for healthcare decision-making. At the start of an intervention, it is often not clear which specific individuals benefit most from a particular intervention alternative or how costs will be distributed on an individual patient level.

**Objective:**

This study aimed at predicting the individual outcome and costs for patients before the start of an internet-based intervention. Based on these predictions, individualized treatment recommendations can be provided. Thus, we expand the discussion of personalized treatment recommendation.

**Methods:**

Outcomes and costs were predicted based on baseline data of 350 patients from a two-arm randomized controlled trial that compared treatment as usual and blended therapy for depressive disorders. For this purpose, we evaluated various machine learning techniques, compared the predictive accuracy of these techniques, and revealed features that contributed most to the prediction performance. We then combined these predictions and utilized an incremental cost-effectiveness ratio in order to derive individual treatment recommendations before the start of treatment.

**Results:**

Predicting clinical outcomes and costs is a challenging task that comes with high uncertainty when only utilizing baseline information. However, we were able to generate predictions that were more accurate than a predefined reference measure in the shape of mean outcome and cost values. Questionnaires that include anxiety or depression items and questions regarding the mobility of individuals and their energy levels contributed to the prediction performance. We then described how patients can be individually allocated to the most appropriate treatment type. For an incremental cost-effectiveness threshold of 25,000 €/quality-adjusted life year, we demonstrated that our recommendations would have led to slightly worse outcomes (1.98%), but with decreased cost (5.42%).

**Conclusions:**

Our results indicate that it was feasible to provide personalized treatment recommendations at baseline and thus allocate patients to the most beneficial treatment type. This could potentially lead to improved decision-making, better outcomes for individuals, and reduced health care costs.

## Introduction

In a clinical context, different forms of behavioral interventions such as face-to-face or internet-based treatments exist for patients with depressive disorders. Clinical and cost effectiveness studies provide important knowledge regarding these treatment alternatives [1]. However, questions remain as to which particular individuals prefer particular treatment types or receive an increased benefit from one specific treatment option over another, especially before the treatment begins. Therapists or other clinicians often make decisions based on personal understanding and experience, leading to high uncertainty or nonoptimal decisions [1]. This uncertainty can potentially result in worse treatment outcomes for individuals and increased health care costs. Simultaneously, policy makers and stakeholders increasingly demand cost-effectiveness evidence in order to support their conclusions and decisions [2].

For supporting these admittedly difficult and complex decisions, approaches exist based on cost analysis or decision analysis [1,3]. The incremental cost-effectiveness ratio (ICER) is a widespread indicator for cost effectiveness [4]. The goal is to support the mentioned decisions by identifying actions that, on average, maximize a specific result [1] such as quality-adjusted life years (QALYs). The ICER is applied on a population level, which means that average values of costs and outcomes are considered for population-level decisions [1,5]. This procedure does not consider any heterogeneity among individuals regarding outcomes and costs. Individual patients, for example, respond differently to treatment and have varying mindsets regarding risks [6,7]. Thus, the average outcomes and costs often do not necessarily represent the best decision for an individual [6]. Even though these aspects are well known, cost-effectiveness analyses based on average values are still widely used [6].

Predictive analyses can provide crucial insight into aspects that influence outcomes and costs of interventions and can be beneficial for patients as well as society [8]. Research that seeks to forecast outcomes for patients with depression already exists. One study, for example, predicted treatment success in the domain of depression and showed that baseline data has predictive power in this context [9]. Another study predicted treatment outcomes of treatment-resistant patients with depression and thereby revealed important predictors such as severity and suicidal risk, among others [10]. These types of statistical procedures can ultimately result in the development of decision support systems in the context of health interventions. In the field of depression treatment, these systems often lead to positive effects and even a reduction of symptoms in various situations [11].

This study focused on making personalized treatment recommendations. For this purpose, we predicted the outcomes and costs for different treatment types, at baseline, on an individual patient level. We applied various machine learning techniques, evaluated them based on their predictive performance, and revealed important features that contributed to the prediction. In order to derive personalized treatment recommendations, we applied an individualized cost-effectiveness analysis based on the ICER. Unlike its traditional utilization based on the ratio of average values, we used individual predictions for each treatment type and its alternative. The predictions and their generated information can provide additional knowledge and enable practitioners, as well as researchers, to individually assign patients at baseline to their most appropriate treatment type in terms of outcomes and costs. This approach is applied to data from an internet-based two-arm randomized controlled trial in the domain of depression.

The forecast of individual outcomes and costs is one of the most important aims in clinical research [12], and personalized analyses and illustrations of cost effectiveness in this context are of increased interest and need [6,13]. Thus, we contribute to existing research by attempting to predict these factors at the start of treatment for each individual and by further proposing a conceptual approach for treatment recommendations, as applied to empirical data.

## Methods

### Data and Preprocessing

The data we utilized originate from the European Union-funded project E-Compared in which the clinical and cost effectiveness of blended treatment (BT) for depression, where internet-based and face-to-face treatments are combined in one integrated treatment protocol, is evaluated and compared with treatment as usual (TAU) in 9 different countries [14]. Participants were aged 18 years or older, met criteria for a major depressive disorder, were not of high suicidal risk, were not being treated for depression, and had access to an internet connection. Table 1 illustrates the different questionnaires used in the study.

The data consisted of individualized information regarding depressive symptoms, medical costs, and other factors. These questionnaires are widely utilized and known and can be found elsewhere [14-18]. The data in the E-Compared project were collected multiple times during the trial: at baseline, 3 months, 6 months, and 12 months. Questionnaires 3, 4, 6, and 7 (according to Table 1) were also available, not only at baseline but also after other times during data acquisition. Because we were interested in recommendations before the start of the actual treatment, we solely used the baseline information as features in this study.

**Table 1:** Data utilized in this study.

Data	Description
Demographic data	N/A^a^
Current treatment	Current treatment type, medication, provider
MINI International Neuropsychiatric Interview	Structured clinical interview for making diagnoses
Quick Inventory of Depressive Symptomatology (16-Item) (Self-Report)	Quick Inventory of Depressive Symptomatology
Patient Health Questionnaire-9	Questions regarding depressive symptoms
5-level EQ-5D	EuroQol questionnaire; measuring generic health status; for calculation of quality-adjusted life years
Costs Associated with Psychiatric Illness	Measurement of healthcare costs and productivity losses
Treatment preferences	Individual preferences for blended treatment or treatment as usual

^a^N/A: Not applicable.

We used QALY as an outcome, as measured by the EuroQol questionnaire (5-level EQ-5D version). Utility weights were calculated using the Dutch tariffs [19]. These weights are a preference-based measure of quality of life anchored at 0 (worst perceivable health) and 1 (perfect health). QALYs were calculated by multiplying the utility weights with the amount of time a participant spent in a particular health state. Transitions between the health states were linearly interpolated. The costs that we aimed to forecast were measured from the societal perspective (including healthcare utilization and productivity losses) based on the adapted version of the Trimbos and Institute for Medical Technology Assessment questionnaires on Costs Associated with Psychiatric Illness [18]. Dutch unit costs were used to value healthcare utilization and productivity losses [20]. Costs for the online part of BT included maintenance and hosting of the treatment and costs that occurred for a therapist to provide feedback to participants. We decided to use costs from a societal perspective because they represent interests of society and all other stakeholder groups [1]. More information on the calculation of the costs can be found elsewhere [21]. As dependent variables, we utilized QALY and costs that appear after a 6-month period. This allowed for more observations compared with the data at 12 months (350 patients vs 212 patients) because not all patients had already finished the treatment process. Because we focused on the outcome data up to 6 months, QALY could have a maximum value of 0.5 in our analysis.

During the data preprocessing phase, we merged all mentioned data from Table 1. This process led to 309 features that could be utilized for the prediction. We then calculated the costs and QALY for each individual. We only included patients for which both dependent variables were not missing. By splitting the dataset into groups for the different treatment types (TAU and BT), some factor levels of an item or feature can go missing. We removed 97 features that had just one level or were missing. Multimedia Appendix 1 lists the omitted items from the questionnaires. The resulting dataset still contained 29,568 missing values. Disregarding these values, and thus deleting them, would lead to a substantial decrease in observations. We therefore utilized two different methods for handling them in order to evaluate which method would perform better regarding the predictive performance. We first imputed the numeric values by sampling from a normal distribution based on the mean value and SD of the corresponding feature. We imputed the categorical predictors by sampling from the categorical distribution of those features. As a second approach, we imputed the missing values by the median (numeric variable) and mode (categorical variable). Finally, we ended up with a dataset of 350 observations (1 for each patient) and 212 features. In the following, we have reported only the results for the latter imputation procedure. In Multimedia Appendix 2, we have also demonstrated the final performances for the first imputation method. However, we decided to utilize the latter method because it led to the best performance in terms of prediction.

### Approach & Statistical Analysis

**Figure 1:**
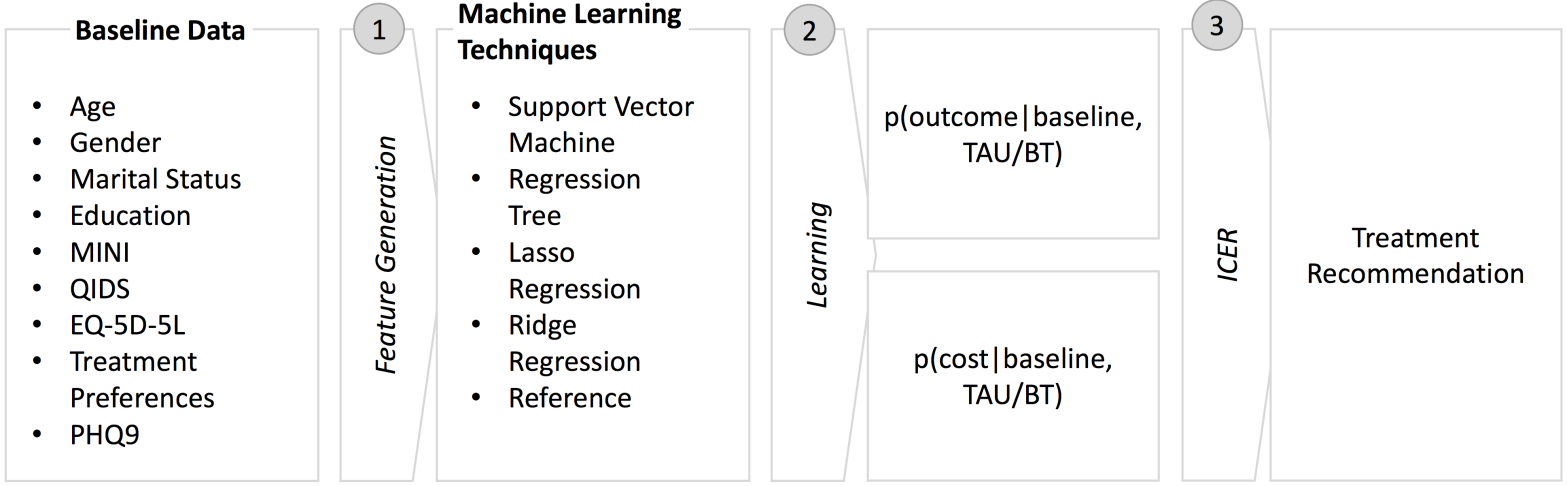
Process for deriving treatment recommendations for individuals. BT: blended treatment; ICER: incremental cost-effectiveness ratio; TAU: treatment as usual.

In order to derive individual treatment recommendations, we utilized the baseline features as input for predicting individual level outcome and costs based on the treatment type, as seen in Figure 1. We applied various machine learning techniques to evaluate which yielded the highest prediction performance. As mentioned by several studies, it is beneficial to compare different statistical procedures in order to eventually find the most precise model, especially when predicting costs due to the challenging nature of this activity [8,22,23]. Because the data consist of numerous features, we applied a feature selection method to reveal variables that contributed to the prediction performance. To demonstrate how the forecasts can be beneficial in recommending treatment types on an individual patient level, we applied the ICER to the predictions.

Specifically, we estimated the conditional probability p(o,c│b,tt) for each treatment type, where *o* is the outcome, *c* is the costs, *b* reflects the baseline features, and *tt* is one of the 2 treatment types. Given the limited amount of data, we assumed that the conditional probability could be factorized as follows: p(o,c│b,tt)=p(o│b,tt)p(c│b,tt).

For the prediction of outcome and costs, we used linear regression and support vector regression (SVR). The latter method has shown good predictive capabilities in various fields [24]. We further utilized regression trees and ridge regression. For finding the optimal parameters, we applied a grid-based search and cross-validation. Additionally, we defined the mean of all outcomes or costs as a reference measure. If unable to achieve a better prediction performance compared with the reference measure, it is questionable if the application of more advanced statistical methods is appropriate in this context. For finding the model that achieves the highest prediction performance, we used leave-one-out cross-validation. That is, one observation is utilized as the test set and the remaining observations are used for training the model. This procedure is repeated for every single observation in the dataset. The error measures we used were root mean square error (RMSE) and mean absolute error (MAE). We have presented both error measures because debate exists as to which measure is more appropriate for the demonstration of predictive performance [25,26].

When utilizing a vast number of features, overfitting presumably occurs. Thus, we used Lasso regression to select features that contributed to the predictive performance. Lasso is a linear regression that introduces a penalty term called regularizer [27]. The error function of the regression, which is to be optimized, consists of the mean square error of the misclassified samples and a term that penalizes the absolute value of the sum of regression coefficients. This linear penalty enforces useless coefficients to shrink toward zero in order to produce a sparse solution. The corresponding optimization problem is illustrated below, where *X* is the baseline feature, *Y* is the outcome or costs, and β is the coefficient: 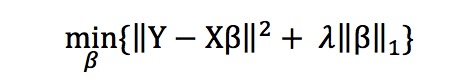
The parameter λ influences the strength of the penalty. Specifically, the higher the value of λ, the higher the penalty. A higher penalty leads to sparser solutions (more coefficients are shrunk to zero). The optimal λ’s are found by utilizing cross-validation. After obtaining the specific features that appear to add to the predictive accuracy, we again predicted the outcome values and costs based on the aforementioned machine learning techniques. This time, however, we only utilized the features that were identified by the Lasso regression. Finally, we selected the algorithm that produced the smallest error and therefore performed best for the outcome and cost predictions. Based on these individual predictions, we calculated the ICER, as seen in the equation: 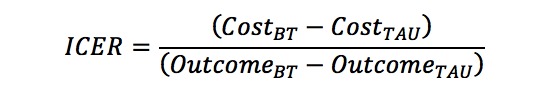


The ICER was then visualized in the cost-effectiveness plane [28]. By predicting the costs and outcomes at baseline and utilizing the ICER, we could then make recommendations about individual patient allocation. We implemented the mentioned models and processes in R (R Core Team; Vienna, Austria) [29].

## Results

### Overall Findings

Before we focused on the outcome and cost predictions, we illustrated the general improvements of the patients for TAU and BT. The E-Compared project hypothesized noninferiority between both treatment types (ie, BT is not less effective) [14]. Improvement was defined as the difference of the start and end value of the cumulated PHQ9 values. The PHQ9 questionnaire is a reliable measure for depression severity [16]. Because we only investigated the improvements for a 6-month period, these results are not final; however, they can indicate a trend. Table 2 shows that the mean baseline score for PHQ9 was 15.35 for BT and 15.42 for TAU. At the 6-month measurement, the scores were 7.85 and 9.49, respectively. Furthermore, 154 patients in the BT group and 140 patients in the TAU group showed improvement. Therefore, we can see that the PHQ9 value decreased more strongly for BT and that the number of improvements for BT exceeded the outcome of TAU. Applying a *t* test for the comparison of the mean end values resulted in the rejection of the hypothesis that both samples had the same mean (*P*=.006).

**Table 2:** Mean of Patient Health Questionnaire-9 scores at baseline and end for treatment as usual and blended treatment as well as the numbers of patients in each condition that improved (N=350).

Measures	Treatment as usual	Blended treatment
Start Patient Health Questionnaire-9, mean	15.42	15.35
End Patient Health Questionnaire-9, mean	9.49	7.85
Patients with improvement, n	140	154
Patients without improvement, n	38	18

**Table 3:** Results for prediction performance based on all baseline features for varying machine learning approaches.

Model	Outcome			Costs in €	
	MAE_O_^a^	RMSE_O_^b^	MAE_C_^c^		RMSE_C_^d^
Support vector regression	0.0714	0.0997	6299.63		9360.50
Regression tree	0.0698	0.0992	6573.94		9406.11
Ridge regression	0.0711	0.1000	6557.69		9187.78
Reference measure	0.0770	0.1017	7024.11		9539.54

^a^MAE_O_: mean absolute error in outcome.

^b^RMSE_O_: root mean square error in outcome.

^c^MAE_C_: mean absolute error in cost.

^d^RMSE_C_: root mean square error in cost.

### Outcome and Cost Prediction

Table 3 illustrates the prediction performance for all utilized machine learning techniques and all baseline features. Overall, the SVR and regression tree had the smallest errors for performance measures. The ridge regression also performed better than the reference measure. Based on a Wilcoxon test, MAEs differed significantly (SVR: *P*_O_=.030, *P*_C_<.001; Tree: *P*_O_=.001, *P*_C_<.001; Ridge: *P*_O_=.049, *P*_C_<.023). Since we had more features than observations, we did not apply ordinary least squares regression when utilizing all baseline features.

We then performed Lasso regression in order to select the important features that contributed to the prediction performance. The tables in Multimedia Appendix 3 show the important features that were utilized and their corresponding coefficient. By applying cross-validation, we chose specific λ values that minimized the mean cross-validated error. For TAU and BT, we used all features up to a λ value of 0.01485 and 0.01479, respectively (433.83 and 651.14 for the cost prediction).

Multiple features appeared repeatedly. Various questions regarding the medication use and the amount of consultations of some kind of therapist, practitioner, or treatment program occurred most often (24 and 16 times, respectively). Furthermore, the anxiety or depression items (6 times), mobility (5 times), origin of the patient (7 times), and energy level questions (4 times) appeared to have an influence on the prediction performance. Using the selected features, we then repeatedly applied the above specified statistical methods in order to achieve a better accuracy.

We observed a general increase in performance (Table 4). All statistical methods performed better than the reference measure (except for RMSE for linear regression and cost prediction), which was again confirmed by a significant Wilcoxon test for MAEs (SVR: *P*_O_<.001, *P*_C_<.001; Regression: *P*_O_<.001, *P*_C_<.001; Tree: *P*_O_=.002, *P*_C_<.001; Ridge: *P*_O_<.001, *P*_C_<.001). This suggested that feature selection resulted in more accurate predictions in this context. The overall results demonstrate *that some machine learning approaches are beneficial when predicting the outcomes and costs*. Since ridge regression predicted the outcome and costs best, we utilized this model in the following analysis.

**Figure 2:**
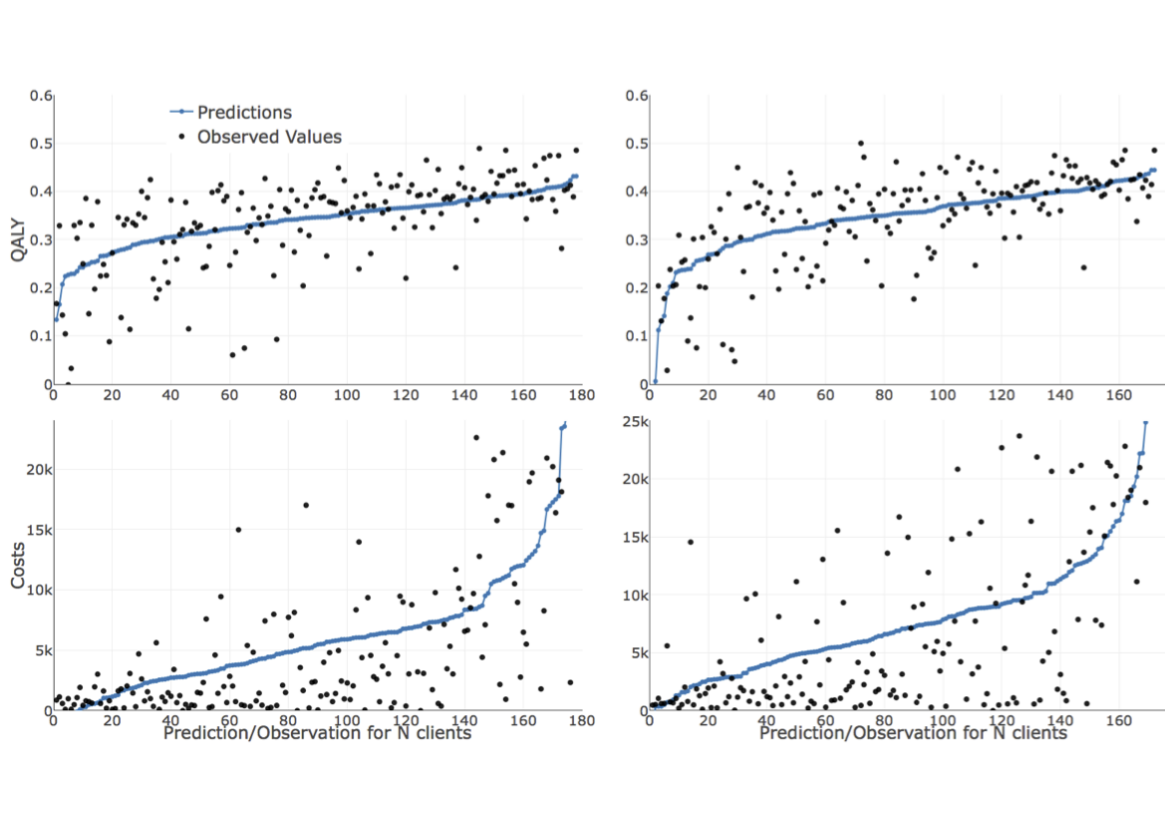
Predicted and observed values for quality-adjusted life years and costs and both treatment types (left panels for treatment as usual and right panels for blended treatment).

Figure 2 illustrates the predicted and observed values for each treatment type and dependent variable (QALY/costs). For estimating the ridge regression penalty term, we implemented 100 cross-validation runs and utilized the parameter that minimized the mean cross-validated error among these runs. The predictions were sorted in an ascending order. The blue markers or lines are the predictions and the black markers are the observed values where the y-axis demonstrates the value of the QALY/costs and the x-axis represents the corresponding patient. We observed that the predicted outcome and costs showed high uncertainty. The broader range of the actual observations around the blue markers for the cost predictions indicated that these were more difficult to achieve than outcome predictions in this context. Visually, however, the trend of the predictions appeared to be as expected, and as illustrated by the increased performance compared with the reference measure; this result indicates a step in the right direction.

**Table 4:** Results for prediction performance based on selected baseline features for varying machine learning approaches.

Model	Outcome		Costs in €	
	MAE_O_^a^	RMSE_O_^b^	MAE_C_^c^	RMSE_C_^d^
Support vector regression	0.0575	0.0812	5164.22	8026.46
Regression	0.0590	0.0793	6436.63	15319.89
Regression tree	0.0684	0.0952	6573.94	9406.11
Ridge regression	0.0553	0.0747	4590.00	6607.31
Reference measure	0.0770	0.1017	7024.11	9539.54

^a^MAE_O_: mean absolute error in outcome.

^b^RMSE_O_: root mean square error in outcome.

^c^MAE_C_: mean absolute error in cost.

^d^RMSE_C_: root mean square error in cost.

**Figure 3:**
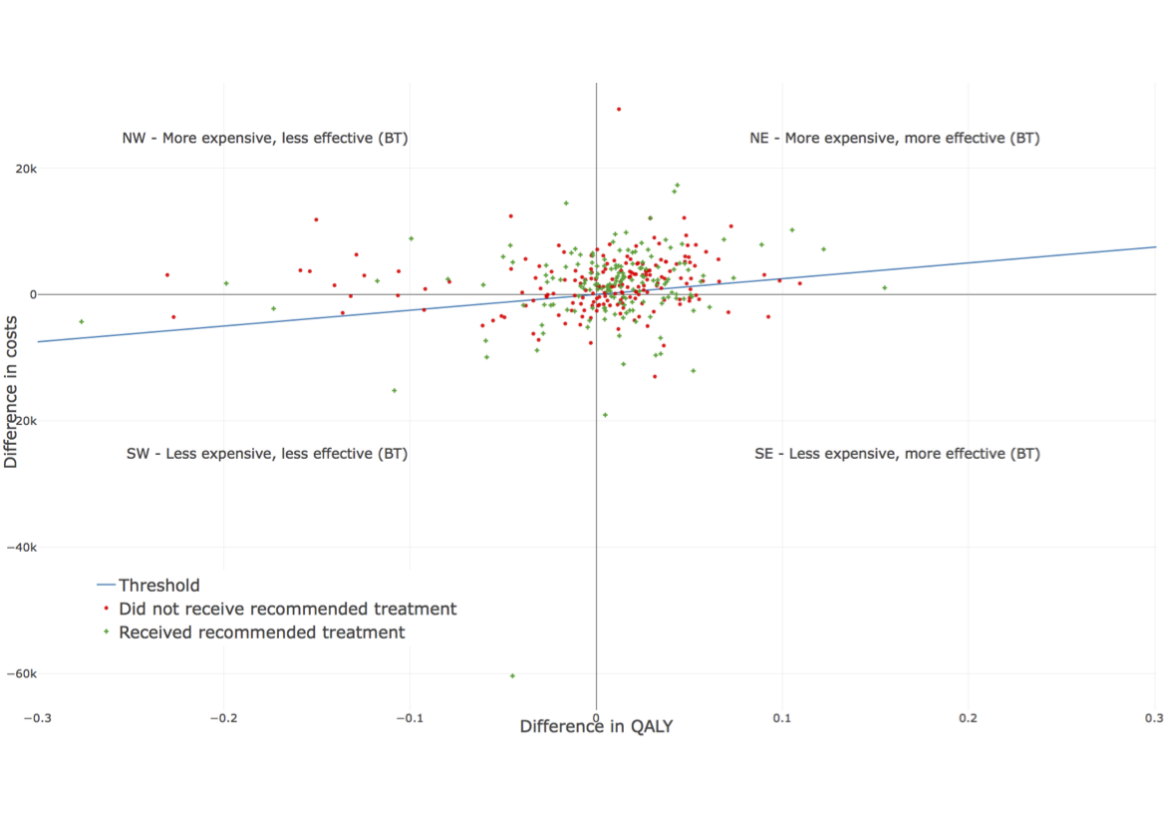
Expected improvement for all patients in relation to costs. The x-axis illustrates the difference in quality-adjusted life years (blended treatment- treatment as usual) and the y-axis the difference in costs (blended treatment- treatment as usual).

### Treatment Recommendation

In order to derive individual treatment recommendations, we represent the differential outcomes and costs in the cost-effectiveness plane, where the y-axis is the difference between the costs of each treatment type and the x-axis is the difference between the clinical effects, as seen in Figure 3 [28]. Each quadrant has a different meaning. In our context, the NE quadrant represents higher costs and positive effects for BT; the SE quadrant indicates that BT is less expensive and more effective (BT dominates); the SW quadrant demonstrates the case where BT is less expensive but less effective; and the NW quadrant displays the situation where BT is more expensive and less effective (TAU dominates) [30]. As a first step, a threshold had to be defined that specified up to which point an additional improvement was worth the costs. In the context of this study, the monetary amount or willingness to pay for gaining one QALY differed by country [30]; the commonly used UK WTP thresholds for QALYs are between 25,000 and 35,000 €/QALY [31]. For this study, we used the conservative estimation of 25,000 €/QALY. A value above this threshold indicated that the treatment type was too expensive. Each patient represented by a green cross received the treatment type we would have recommended based on the prediction.

On the contrary, each patient that had a red circle should have received the other treatment type based on the forecasts. Questionnaire items that deviate tremendously for either TAU or BT create high differences when calculating the ICER. The point for the participant at the bottom of Figure 3 at (−0.04, −60.420), for example, is due to the fact that this patient reported a large number of hospital admissions. Since these are very expensive, it led to very high costs for this particular patient, and thus, the difference in costs between BT and TAU was high. Following this process, it is possible to recommend the likely most beneficial treatment type, on an individual level, at baseline.

Table 5 is a contingency table consisting of the patients for whom we recommended a specific treatment type. Only 46.57% (163/350) of all patients were treated using the treatment type we would recommend based on our models and the particular ICER threshold.

We then calculated potential outcomes and costs on a population level assuming the patients would have been allocated according to the predictions. For patients who had already received the recommended treatment type, we utilized the observed outcomes and costs. For patients for whom the actual treatment type was not recommended, we utilized the predictions of the model. Then, QALYs would have decreased by 1.98%, while at the same time, a reduction in costs of 5.42% could have been achieved.

**Table 5:** Treatment recommendation for all patients (N=350).

Treatment type	Recommended blended treatment, n (%)	Recommended treatment as usual, n (%)
Received blended treatment	70 (20)	102 (29.14)
Received treatment as usual	85 (24.29)	93 (26.57)

## Discussion

### Principal Findings

Given the growth in demand for personalized treatments and the need for a reduction in costs, predictions of outcomes and costs, in the context of mental health, are increasingly important [3]. In this study, we proposed an approach for personalized treatment recommendations at baseline. Here, individuals are assigned to the most beneficial treatment *before treatment*, which can, if desired, even be automated. We derived these recommendations by predicting patient individual QALYs and costs based on data from a European Union-funded project. We then used the ICER and the cost-effectiveness plane as an individualized treatment recommendation tool. Nowadays, decisions are often made based on the ICER; we proposed a feasible path that allows the individualization and tailoring of this process.

We illustrated that the utilization of all baseline features is not necessarily appropriate in this context. Taking advantage of feature selection techniques can increase prediction performance. As a result, we found that consultations with some kind of therapist, medication usage, anxiety or depression information (severity), mobility items (ie, “I have no problems in walking about”), and origin of the patient play an important role when predicting outcomes and costs in the context of digital health interventions. Therefore, including questionnaires that contain these factors and subsequently utilizing these features in statistical analyses when predicting outcomes and costs can be beneficial. We further illustrated that experimentation with different statistical methods benefits the final results since considerable varying performances occurred among the methods.

However, we demonstrated that prediction is a challenging task. Even though the results suggest that predictive power exists in the baseline features, our analyses indicated that the predictions, and thus the recommendations, come with uncertainty when only baseline information is available. In general, the predictive uncertainty is due to two sources. The first source is the uncertainty in the estimated parameters. With an increased amount of data, the uncertainty in parameter estimation reduces. This does not mean that we would achieve perfect predictions because the second source is related to the variance of treatments that cannot be explained by the model. More specifically, the models do not fully represent the reality and all its complexity. Hence, although the estimation of the model parameters improves with more data, the uncertainty that results from the model specifications and inability of the baseline information to precisely predict results remains. Nevertheless, we showed that we were able to predict the outcomes and costs better, compared with using the mean of the dependent variables as prediction (reference measure). Therefore, we are convinced that the baseline features do include some information regarding the forecast of outcomes and costs and can support practitioners in their decision-making process. Thus, combining these results with the ICER enabled us to provide treatment recommendations on an individual level.

As mentioned earlier, if the patients would have been allocated according to our predictions, QALYs would have decreased by 1.98% and a simultaneous reduction in costs of 5.42% could have been achieved. These results are based on a specific ICER threshold. When applying this procedure in a real-world setting, this threshold can be adjusted to values set by experts or policy makers or available budgets. These experts must make decisions regarding the monetary resources they would want to spend on a specific QALY gain. Thus, the outcome and costs can be controlled by setting this threshold. As suggested by a previous study [32], the cost-effectiveness decision rule might be modeled in a nonlinear form. For example, the value of improvements may vary among the outcome levels. Particularly, a difference between 0.1 and 0.2 on the scale might be more important than a difference between 0.8 and 0.9, even though the absolute difference is the same. The absolute severity of the symptoms can also play an additional role in this context. It might not be justifiable to spend additional monetary effort if a specific patient already does not suffer from severe symptoms. Therefore, experts in the field need to choose appropriate values for the ICER threshold based on their experiences and knowledge and even consider a nonlinear specification.

Even though these results are preliminary, the implementation of such predictive models in clinical decision support systems for usage in interventions can be beneficial. We envision developing a system that incorporates these models and provides treatment recommendations for individuals. However, investment into other aspects is necessary for the realization of such support systems. Besides the technical implementation, the creation of information systems in this context also requires interdisciplinary collaboration among clinicians, computer scientists, and other decision makers [33]. Future users, for example decision makers or therapists, need to be educated appropriately and also be involved in the design phase of the system and its requirements and development, while at the same time, the IT specialists need to be confronted with content-related issues of the user [34,35]. Thus, implementation should be carefully planned and considered as organizational development [36]. Furthermore, a vast amount of financial and organizational resources can be required for the implementation [33], and clinical decision makers need to understand the value and limitations of such decision support systems. Additionally, we need to be cautious with the interpretability of the results because in individual cases, recommendations might lead to suboptimal outcomes and high uncertainty depending on the particular context. Overall, these systems may be used in the future to support the decision-making process of clinicians and therapists and not to replace their treatment recommendations.

### Limitations

This study has certain limitations. One limitation is the fact that we utilized data after a 6-month period. Usually, the preferred outcome for cost-effectiveness analysis is based on 12 months. Another limitation, which is closely associated with the previous aspect, is the size of the dataset we used. Given the complexity of the problem, it is inevitable that variations in performance occur when predicting other datasets. Thus, for achieving higher accuracy in predictions, obtaining more data is crucial. Even though our results are promising, more data and evaluations are needed in order to investigate the generalizability of these outcomes and improve the predictive accuracy of statistical techniques. Besides the size of the dataset, the data are heterogeneous in different ways. For example, the data were collected from 9 different European countries, with each having their own country-specific conditions [14]. This can result in country-specific patterns in the data. Given the limited amount of observations on a national level, we have not explored this multi-level structure. Additionally, the dataset consists of a large amount of missing values that needed imputation. Making all baseline questions mandatory for the patients can lead to an increased performance of the statistical procedures and can therefore lower uncertainty.

### Conclusions

This study investigated how patients can be allocated to different treatment types in order to increase clinical and cost effectiveness. We demonstrated how to predict outcomes and costs in this context and proposed an approach for individualized treatment recommendations by utilizing the ICER. Simultaneously, we evaluated a variety of machine learning techniques and demonstrated specific features that contribute to the prediction performance. The results are indicative of progress. We hope that policy makers increasingly understand the benefit of predictive modeling in this context and apply these types of models to make better and simultaneously more personalized treatment choices. We further hope that we can contribute to the decision-making process in this field by providing a path that allows the prediction of eventual outcomes and costs on an individual basis before the onset of treatment.

## Appendix

Multimedia Appendix 1 Omitted items from analysis.

Multimedia Appendix 2 Results for prediction performance based on sampling from normal and categorical distribution for varying machine learning approaches.

Multimedia Appendix 3 Important baseline features based on Lasso regression for quality-adjusted life years and cost prediction for treatment as usual and blended treatment.

## Abbreviations

BT: blended treatment
ICER: incremental cost-effectiveness ratio
MAE: mean absolute error
QALY: quality-adjusted life years
RMSE: root mean square error
SVR: support vector regression
TAU: treatment as usual
