# Supplementary material for: Predicting Therapy Success and Costs for Personalized Treatment Recommendations Using Baseline Characteristics: Data-Driven Analysis
Source: J Med Internet Res. 2018 Aug 21;20(8):e10275. doi: 10.2196/10275 (PMC6123535; doi:10.2196/10275)
Supplement: Multimedia Appendix 1 [file jmir_v20i8e10275_app1.pdf]

Multimedia Appendix 1

Omitted Items:

Item No.	Item Description
1	Do you have access to a fast Internet connection (apref2)
2	Treatment program at other institution 1/2/3/4 (aTicp5d1/aTicp5e1/aTicp5f1/aTicp5g1)
6	Other primary care 1/2/3/4/5 (aTicp1i1/aTicp1j1/aTicp1h1/aTicp1g1/aTicp1k1)
11	How many times did you consult other primary care 4/5 (aTicp1j2/aTicp1k2)
13	Number of days a day-time treatment program (institution 2/3/4) (aTicp5e2/aTicp5f2/aTicp5g2)
16	Number of parts of days a part-time treatment program (institution 2/3/4) (aTicp5e3/aTicp5f3/aTicp5g3)
19	Other Tranquilizers or sleep medication (aTicp27/aTicp28)
21	Other mental care 1/2/3/4 (aTicp2h1/aTicp2i1/aTicp2j1/aTicp2k1)
25	Other complementary by name 1/2/3/4/5 (aTicp3f1/aTicp3g1/aTicp3h1/aTicp3i1/aTicp3j1)
30	How many times did you consult other complementary therapists 2/3/4/5 (aTicp3g2/aTicp3h2/aTicp3i2/aTicp3j2)
34	Received domestic care, number of months (aTicp9a)
35	Other provider of treatment (atreat15b)
36	Medication use (period Amitryptiline (Tryptizol) (aTicp11d1)
37	How long have you been in treatment (atreat15c)
38	Medication use (dosage Nortriptyline (Nortrilen) (aTicp16b)
39	Other type of treatment (atreat17a)
40	Medication use (Frequency Nortriptyline (Nortrilen) (aTicp16c)
41	Depressive episode current (amini1)
42	Medication use (period Nortriptyline (Nortrilen) (aTicp16d1)
43	Specify drugs taken (amini20b)
44	Antidepressants (aTicp20)
45	Specify drugs (amini20d)
46	Other antidepressants (aTicp21)
47	Psychotic disorder current (amini21)
48	Period other depressants (aTicp21d1)
49	Self care (aEQ5D5L2)
50	Pain/Discomfort (aEQ5D5L4)
51	How many times did you consult the Speech therapist (aTicp1d)
52	Times of rehabilitation clinic admissions (aTicp6c1)
53	Other medication for mental health complaints (aTicp29)
54	Medication use (other period Amitryptiline (Tryptizol)) (aTicp11d2)
55	Nights of rehabilitation clinic admissions (aTicp6c2)
56	Medication use (other medications for mental health complaints) (aTicp30)
57	Medication use (period Flurazepam (Dalmadorm)) (aTicp25d1)
58	How many times did you consult other mental care 4 (aTicp2k2)
59	Type of other institution at which admissions 1 (aTicp6e1)
60	Period other medication for mental health complaints (aTicp30d1)
61	How long have you been taking antipsychotic medication (atreat8)
62	Type of other institution at which admissions 2 (aTicp6f1)
63	Other Treatment (atreat2b)
64	How long have you been taking this other medication (atreat12)
65	Times of other institution 2 admissions (aTicp6f2)
66	Anti-depressants (atreatMed)
67	Who is delivering the psychotherapy (atreat14a)

68	Nights of other institution admissions (aTicp6f3)
69	Other provider of anti-depressants (atreat5a)
70	Recency of hypomanic episode (amini6b)
71	Type of other institution at which admissions 3 (aTicp6g1)
72	Who is providing the tranquillizers (atreat7)
73	Abuse non-alcohol psychoactive substance use disorder current (amini20a)
74	Times of other institution admissions (aTicp6g2)
75	Other provider of antipsychotic medication (atreat9a)
76	Abuse non-alcohol psychoactive substance current (amini20c)
77	Nights of other institution admissions (aTicp6g3)
78	Other provider of sleep medication (atreat11a)
79	Depressive episode lifetime (amini23)
80	Received nurse care, number of months, in last 3 months (aTicp7a)
81	Other provider of this other medication (atreat13a)
82	Who is delivering the other treatment (atreat15a)
83	Received daily care, number of months (aTicp8a)
84	Other provider of psychotherapy (atreat14b)
85	Received daily care, number of hours per week (aTicp8b)
86	Thoughts of Death or Suicide (aQIDS12)
87	Medication use (Other medication for mental health complaints) (aTicp30a)
88	Medication use (Nortriptyline (Nortrilen)) (aTicp16a)
89	How many times did you consult the Holistic therapist (aTicp3c)
90	Medication use (Amitryptiline (Tryptizol)) (aTicp11a)
91	Marital status (aMarital)
92	Hypomanic episode (amini6a)
93	How many times did you consult the Natural healer (aTicp3e)
94	How many times did you consult the Professional from a clinic for alcohol and drugs or similar institution (aTicp2f)
95	Who is providing the anti-depressants (atreat5)
96	How long have you been taking tranquillizers (atreat6)
97	Who is providing this other medication (atreat13)
