# Supplementary material for: Predicting Therapy Success and Costs for Personalized Treatment Recommendations Using Baseline Characteristics: Data-Driven Analysis
Source: J Med Internet Res. 2018 Aug 21;20(8):e10275. doi: 10.2196/10275 (PMC6123535; doi:10.2196/10275)
Supplement: Multimedia Appendix 2 [file jmir_v20i8e10275_app2.pdf]

Multimedia Appendix 2

Results for prediction performance based on sampling from normal and categorical distribution and all baseline features for varying machine learning approaches (MAE: mean absolute error, RMSE: root mean square error):

**Model**	**Outcome**		**Costs in €**	
	MAE_O	$RMSE_O$	MAE_C	$RMSE_C$
SVR	0.0737	0.1014	6539.97	9466.11
Regression Tree	0.0765	0.1040	6471.05	9346.40
Ridge Regression	0.0647	0.0870	6044.44	8395.56

Results for prediction performance based on sampling from normal and categorical distribution and selected baseline features for varying machine learning approaches (MAE: mean absolute error, RMSE: root mean square error):

**Model**	**Outcome**		**Costs in €**	
	MAE_O	$RMSE_O$	MAE_C	$RMSE_C$
SVR	0.0598	0.0838	5195.46	8211.82
Regression	0.0599	0.0794	5204.89	7194.91
Regression Tree	0.0707	0.0958	6229.68	9278.88
Ridge Regression	0.0565	0.0746	5001.75	6921.86
