# Supplementary material for: Predicting Therapy Success and Costs for Personalized Treatment Recommendations Using Baseline Characteristics: Data-Driven Analysis
Source: J Med Internet Res. 2018 Aug 21;20(8):e10275. doi: 10.2196/10275 (PMC6123535; doi:10.2196/10275)
Supplement: Multimedia Appendix 3 [file jmir_v20i8e10275_app3.pdf]

Multimedia Appendix 3

Important baseline features based on Lasso regression for TAU (including single levels for each item) and QALY prediction for λ=0.01485:

Feature	Parameter coefficient
(Intercept)	3.897*e*−1
Cumulated PHQ value (aPHQScore)	-3.013*e*−3
Anxiety/Depression (I am slightly anxious or depressed) (aEQ5D5L5)	2.898*e*−2
How many times did you consult the General practitioner (aTicp1a)	-1.732*e*−3
Mobility (I have no problems in walking about) (aEQ5D5L)	1.502*e*−2
General interest (I have virtually no interest in the activities I used to enjoy) (aQIDS13)	-6.159*e*−3
Usual activities (I have severe problems doing my usual activities) (aEQ5D5L3)	-1.440*e*−2
Anxiety/Depression (I am severely anxious or depressed) (aEQ5D5L5)	-9.663*e*−3
How many times did you consult other primary care (aTicp1h2)	-3.168*e*−3
Medication use (other period other depressants 1) (aTicp20d2)	-3.183*e*−3
How many times: medical specialist at an outpatient clinic (aTicp4)	-2.726*e*−3
Medication use (dosage Venlafaxine (Efexor)) (aTicp19b)	-7.900*e*−5
Medication use (Frequency other depressants 1) (aTicp20c)	-1.710*e*−3
Agoraphobia current (yes) (amini11)	-1.263*e*−3
Age at baseline (aAge)	-5.200*e*−5

Important baseline features based on Lasso regression for BT (including single levels for each item) and QALY prediction for λ=0.01479:

Feature	Parameter coefficient
(Intercept)	2.930*e*−1
Mobility (I have severe problems in walking about) (aEQ5D5L1)	-1.760*e*−1
Mobility (I have no problems in walking about) (aEQ5D5L1)	3.180*e*−2
Cumulated PHQ value (aPHQScore)	-2.220*e*−3
Energy level (I really cannot carry out most of my usual daily activities because I just do not have the energy) (aQIDS14)	-1.863*e*−2
Anxiety/Depression (I am severely anxious or depressed) (aEQ5D5L5)	-1.830*e*−2
Usual activities (I have no problems doing my usual activities) (aEQ5D5L3)	1.630*e*−2
Usual activities (I have severe problems doing my usual activities) (aEQ5D5L3)	-1.806*e*−2
How many times did you consult other mental care 3 (aTicp2j2)	-5.045*e*−3
Medication use (period Other medication for mental health complaints) (other) (aTicp29d1)	7.260*e*−2
How many times did you consult the general practitioner (aTicp1a)	-2.890*e*−4
Trouble concentrating on things, such as reading the newspaper or watching television (Nearly every day) (aPHQ07)	-5.277*e*−3
How many times did you consult the Dietician (aTicp1e)	-1.050*e*−2
Who is providing the sleep medication (Psychiatrist) (atreat11)	2.706*e*−3
Medication use (dosageFluoxetine (Prozac)) (aTicp14b)	-5.700*e*−5

Important baseline features based on Lasso regression for TAU (including single levels for each item) and cost prediction for λ=433.83:

Feature	Parameter coefficient
(Intercept)	1.170*e+6*
Little interest or pleasure in doing things (Several days) (aPHQ01)	-5.675*e+2*
Trouble falling or staying asleep, or sleeping too much (Nearly every day) (aPHQ03)	1.401*e+3*
Do you have a preference for one of the treatments offered (No preference) (apref1)	-3.972*e+2*
Do you have a preference for one of the treatments offered (Treatment as usual not including the online treatment) (apref1)	-3.972*e+2*
Mobility (I have no problems in walking about) (aEQ5D5L1)	-8.392*e+2*
Mobility (I have slight problems in walking about) (aEQ5D5L)	2.145*e+2*
Anxiety/Depression (I am severely anxious or depressed) (aEQ5D5L5)	1.112*e+3*
Anxiety/Depression (I am slightly anxious or depressed) (aEQ5D5L5)	-4.171*e+2*
How many times did you consult the general practitioner (aTicp1a)	6.831*e+1*
How many times did you consult a therapist for physical therapy (aTicp1b)	2.048*e+1*
How many times did you consult the Dietician (aTicp1e)	4.193*e+1*
How many times did you consult other primary care 1 (aTicp1g2)	5.988*e+2*
How many times did you consult the Psychiatrist (aTicp2d)	1.565*e+2*
How many times did you consult other mental care 1 (aTicp2h2)	-9.298*e+1*
How many times did you consult the Acupuncturist (aTicp3a)	5.166*e+1*
How many times: medical specialist at an outpatient clinic (aTicp4)	2.459*e+2*
Times of other institution admissions (aTicp6e2)	-4.074*e+2*
Medication use (dosage Citalopram (Cipramil)) (aTicp12b)	2.421*e+1*
Medication use (other periodCitalopram (Cipramil)) (aTicp12d2)	-3.800*e+1*
Medication use (Fluoxetine (Prozac)) (aTicp14a)	-6.932*e+2*
Medication use (other period Nortriptyline (Nortrilen)) (aTicp16d2)	2.518*e+3*
Medication use (other) (aTicp17d1)	-2.363*e+3*
Medication use (dosage other depressants 1) (aTicp20b)	6.129*e+1*
Medication use (Frequency Oxazepam (Seresta)) (aTicp22c)	-9.576*e+1*
Medication use (period Oxazepam (Seresta)) (aTicp22d1)	-1.410*e+3*
Medication use (period Zopiclon (Imovane)) (aTicp26d1)	1.424*e+3*
Medication use (dosage Other Tranquilizers or sleep medication) (aTicp27b)	8.460*e+0*
Medication use (other period for Other medication for mental health complaints) (aTicp29d2)	-4.046*e+2*
Do you have a paid job (yes) (aTicp39)	1.052*e+3*
How many hours does your contract specify (aTicp40)	3.720*e+1*
Did health problems oblige you to call in sick from work at any time (Yes, I was off work during the full three months) (aTicp42)	4.242*e+3*
On which date did you call in sick from work first because of health problems (aTicp43)	-8.500*e-5*
On how many working days did you call in sick from work because of health problems in the past three months (aTicp45)	5.469*e+1*
Was your job performance adversely affected by health problems (yes) (aTicp46)	7.219*e+2*
Rate how well performed on days bothered by health problems (aTicp48)	-1.439*e+2*
What type of treatment do you receive (Medication) (aTreat2a)	-8.896*e+0*
How long have you been taking sleep medication (1-6 months) (atreat10)	-2.746*e+3*
How long have you been taking sleep medication (More than 1 year) (atreat10)	7.832*e+2*
Who is providing the sleep medication (Psychiatrist) (atreat11)	-6.851*e+2*
What type of treatment did you receive (Medication) (atreat17)	-7.687*e+2*

Suicidal risk current (yes) (amini5a)	5.668*e+2*
Recency manic episode (Lifetime) (amini7b)	3.520*e+1*
Agoraphobia current (yes) (amini11)	-1.532*e+3*
Panic disorder without agoraphobia current (amini12)	4.787*e+2*
Panic disorder with agoraphobia current (yes) (amini13)	-3.185*e+3*
Obsessive compulsive disorder current (yes) (amini16)	-7.251*e+2*
Falling asleep (I take at least 30 minutes to fall asleep, some nights) (aQIDS01)	-2.655*e+1*
Increased Appetite (I regularly eat more often and or greater amounts of food than usual) (aQIDS07)	6.115*e+2*
Weightloss (I have lost 2.5 kilos or more) (aQIDS08)	-6.544*e+2*
Weightgain (I have gained 2.5 kilos or more) (aQIDS09)	1.150*e+3*
Weightgain (I have not had a change in my weight) (aQIDS09)	1.029*e+1*
Concentration/Decision Making (Most of the time, I struggle to focus my attention or to make decisions) (aQIDS10)	-7.034*e+1*
Energy level (I have to make a big effort to start or finish my usual daily activities) (aQIDS14)	7.805*e+1*
Energy level (I really cannot carry out most of my usual daily activities because I just do not have the energy) (aQIDS14)	1.160*e+3*
Country code (Germany) (cc)	4.782*e+2*
Country code (Poland) (cc)	-2.842*e+3*
Country code (Netherlands) (cc)	4.058*e+3*
Country code (Spain) (cc)	-7.364*e+2*
Country code (UK) (cc)	4.058*e+3*

Important baseline features based on Lasso regression for BT (including single levels for each item) and cost prediction for λ=651.14:

**Feature**	**Parameter coefficient**
(Intercept)	-1.524*e+6*
Little interest or pleasure in doing things (Not at all) (aPHQ01)	-5.829*e+2*
Trouble falling or staying asleep, or sleeping too much (Several days) (aPHQ03)	-2.313*e+2*
Poor appetite or overeating (Nearly every day) (aPHQ05)	1.742*e+3*
Trouble concentrating on things, such as reading the newspaper or watching television (Not at all) (aPHQ07)	-5.504*e+2*
Willing to carry a Smartphone delivered by treatment team (no) (apref4)	-1.444*e+3*
Usual activities (I have no problems doing my usual activities) (aEQ5D5L3)	-2.353*e+1*
Anxiety/Depression (I am moderately anxious or depressed) (aEQ5D5L5)	1.555*e+2*
How many times did you consult the industrial physician (aTicp1f)	5.155*e+2*
How many times did you consult other primary care 1 (aTicp1g2)	7.368*e+2*
How many times did you consult other mental care 3 (aTicp2j2)	1.621*e+2*
How many times did you consult the Acupuncturist (aTicp3a)	9.675*e+2*
Nights of regular hospital admissions (aTicp6a2)	4.013*e+1*
Times of other institution admissions (aTicp6e2)	-2.483*e+2*
Medication use (Citalopram (Cipramil)) (aTicp12a)	-7.973*e+2*
Nights of regular hospital admissions (aTicp15d2)	-3.801*e+2*
Medication use (dosage Venlafaxine (Efexor)) (aTicp19b)	1.879*e+1*
Medication use (period Venlafaxine (Efexor)) (aTicp19d1)	1.692*e+3*
Medication use (dosage other depressants 1) (aTicp20b)	1.490*e+1*
Medication use (other antidepressant 2) (yes) (aTicp21a)	1.850*e+3*

Medication use (dosage other depressants 2) (aTicp21b)	2.358*e+2*
Medication use (other period Oxazepam (Seresta)) (aTicp22d2)	4.460*e+2*
Medication use (dosage Other medication for mental health complaints) (aTicp30b)	1.348*e+3*
Do you have a paid job (yes) (aTicp39)	2.453*e+3*
How many hours does your contract specify (aTicp40)	5.939*e+0*
Job questions: over how many days are these hours distributed (aTicp41)	1.562*e+2*
Did health problems oblige you to call in sick from work at any time (Yes, I was off work during the full three months) (aTicp42)	1.008*e+4*
On which date did you call in sick from work first because of health problems (aTicp43)	1.120*e-4*
On how many working days did you call in sick from work because of health problems in the past three months (aTicp45)	1.614*e+2*
Number of hours you had to catch up on work unable to perform (aTicp49b)	5.345*e+0*
How long have you been in psychotherapy (6 months-1 year) (atreat14c)	1.191*e+3*
How long have you been in psychotherapy (Less than one month) (atreat14c)	-1.830*e+10*
What type of treatment did you receive (Psychotherapy) (atreat17)	2.619*e+3*
Falling asleep (I take at least 30 minutes to fall asleep, some nights) (aQIDS01)	9.567*e+2*
General interest (There is no change from usual in how interested I am in other people or activities) (aQIDS13)	-1.854*e+3*
Energy level (I really cannot carry out most of my usual daily activities because I just do not have the energy) (aQIDS14)	2.764*e+1*
Feeling Restless (I do not feel restless) (aQIDS16)	9.588*e+2*
Country code (Germany) (cc)	2.794*e+2*
Country code (UK) (cc)	-2.967*e+2*
